# Mapping Variability of Mycotoxins in Individual Oat Kernels from Batch Samples: Implications for Sampling and Food Safety

**DOI:** 10.3390/toxins17010034

**Published:** 2025-01-11

**Authors:** Irene Teixido-Orries, Francisco Molino, Bianca Castro-Criado, Monika Jodkowska, Angel Medina, Sonia Marín, Carol Verheecke-Vaessen

**Affiliations:** 1 Applied Mycology Unit, Department of Food Technology, Engineering and Science, AGROTECNIO-CERCA Centre, University of Lleida, Av. Rovira Roure 191, 25198 Lleida, Spain; irene.teixido@udl.cat (I.T.-O.); francisco.molino@udl.cat (F.M.); biancateresa.castro@udl.cat (B.C.-C.); sonia.marin@udl.cat (S.M.); 2 Magan Centre of Applied Mycology, Cranfield University, Cranfield MK43 0AL, UK; m.jodkowska@cranfield.ac.uk (M.J.); a.medinavaya@cranfield.ac.uk (A.M.)

**Keywords:** mycotoxins, *Fusarium*, deoxynivalenol, individual oat kernel, co-occurrence, ratios, kernel weight, contamination heterogeneity, legislation compliance, sampling

## Abstract

Oats are susceptible to contamination by *Fusarium* mycotoxins, including deoxynivalenol (DON), zearalenone (ZEN), and T-2/HT-2 toxins, posing food safety risks. This study analyses the variation in levels of 14 mycotoxins in 200 individual oat kernels from two DON-contaminated batch samples (mean = 3498 µg/kg) using LC-MS/MS. The samples also contained deoxynivalenol-3-glucoside (DON-3G), 3-acetyldeoxynivalenol (3-ADON), 15-acetyldeoxynivalenol (15-ADON), and ZEN. Contamination levels varied notably among individual kernels, with DON detected in 70% of them, followed by DON-3G (24.5%) and 3-ADON (20.5%). Importantly, 8% of kernels exceeded the EU legal limit for DON (1750 µg/kg), and some occasionally surpassed limits for ZEN and T-2/HT-2. Correlation analyses revealed strong associations between DON and its derivatives but weaker correlations with other toxins. Mycotoxin ratios varied widely, indicating that although they often co-occur, their concentrations differ between kernels. Contamination did not significantly impact kernel weight, though a slight trend toward lower weights in contaminated kernels was noted. Additionally, sampling statistics showed that as the percentage of selected kernels increased, the probability of batch sample rejection for DON contamination rose significantly. The study highlights the heterogeneity of mycotoxin contamination in oat batches, emphasising the importance of accurate detection and regulatory compliance to ensure safer oat-based products.

## 1. Introduction

Oat (*Avena sativa* L.) is a relevant crop in many northern hemisphere countries, traditionally used primarily as animal feed. Recently, oats have gained popularity for human consumption due to their valuable nutritional properties and multifunctional characteristics [1]. This versatility has led to an increase in the production and consumption of oat-based products, which not only enhance nutritional value but also contribute significantly to consumer health [2].

Nevertheless, oats are highly susceptible to Fusarium Head Blight (FHB), one of the most significant cereal diseases worldwide [3]. This disease, caused by various *Fusarium* species, can infect and damage cereal heads and grains in the pre-harvest stage, severely affecting yield and quality. *Fusarium* spp. produce a range of mycotoxins during infection, which are secondary metabolites toxic to humans and animals when ingested through contaminated cereals or cereal-based products [4]. Consequently, *Fusarium* mycotoxins represent a critical safety concern for cereal grains intended for food and feed, including oats. Key *Fusarium* species found in oats include *F. graminearum*, *F. culmorum*, *F. langsethiae*, and *F. poae*, all of which produce various mycotoxins, including trichothecenes (types A and B) and zearalenone (ZEN) [5,6].

Among these, type A trichothecenes include toxins such as T-2 toxin (T-2), HT-2 toxin (HT-2), diacetoxyscirpenol (DAS), and 15-acetoxyscirpenol (15-AS), while type B trichothecenes include, among others, deoxynivalenol (DON), 3-aceyldeoxynivalenol (3-ADON), and 15-acetyldeoxynivalenol (15-ADON). Some mycotoxins can transform, forming deoxynivalenol-3-glucoside (DON-3G) or T-2 triol and T-2 tetraol [7]. Trichothecenes ingestion can cause gastrointestinal distress, skin irritation, immunosuppression, and haematological effects [8]. Additionally, other fungi such as *Aspergillus* and *Penicillium* can infect oats pre-harvest or post-harvest, producing mycotoxins like ochratoxin A (OTA), ochratoxin B (OTB), or ochratoxin-alpha (OTα), which can cause kidney damage and immunosuppression and are potentially carcinogenic [9]. Among all these mycotoxins, DON is the most common in oats in the European Union (EU) [5,10], Canada [11], and Norway [12]. T-2 and HT-2 also have a high incidence in oats, and they have become a food safety concern in Norway [3], Ireland [13], and the United Kingdom [14,15].

To safeguard human and animal health, the EU established maximum limits for DON, ZEN, OTA, and the sum of T-2 and HT-2 in unprocessed oat batches [16,17,18], set at 1750 µg/kg, 100 µg/kg, 5 µg/kg, and 1250 µg/kg, respectively. Moreover, the European Commission has highlighted the importance of considering not just the content of DON but the sum of DON, 3-ADON, 15-ADON, and DON-3G to assess the human health risk [18]. Regarding sampling, European Regulation 2023/2782 outlines the sampling methodology for mycotoxins in cereal batches [19]. However, existing sampling methods for detecting mycotoxins face significant limitations, such as non-representative sampling and potential underestimation of contamination levels [20,21]. These issues arise from variability in mycotoxin levels among individual cereal kernels within batch samples caused by factors like uneven fungal spore distribution and microclimates within the field [22]. This variability can lead to batch misclassification, complicating regulatory compliance and quality control, making it crucial for accurate risk assessment of mycotoxin exposure [23]. Nonetheless, this variance in the concentration of mycotoxins can be reduced by applying good sampling principles, including using proper tools and procedures [21].

Although the contamination of certain mycotoxins in individual grains in batches has been studied in other cereals, it has never been investigated for oat grains. Therefore, there is a lack of research on mapping the intra-lot variability of mycotoxins in individual oat kernels. Studies in wheat, barley, and maize have shown that a minority of kernels with high mycotoxin contamination can be responsible for a whole batch rejection in the industry based on sampling and analysis steps [21,22,24]. Therefore, an effective mitigation strategy would be the identification and removal of highly contaminated cereal kernels from batches before industrial processing, reducing overall contamination and avoiding batch rejection, contributing to economic efficiency and sustainability in the food industry [24]. Consequently, research on mycotoxin variability in individual oat kernels can help improve sampling methods, leading to more accurate assessments of mycotoxin contamination, appropriate regulatory practices, and enhanced consumer safety.

For those reasons, the present study aims to increase the understanding of mycotoxin levels in individual oat kernels, principally *Fusarium* mycotoxins. We assess the variability of these levels across two batch samples with high content of *Fusarium* mycotoxins and analyse the implications for sampling methods and food safety. Additionally, we investigate correlations, co-occurrence, and ratios between mycotoxins in each kernel. The complete list of mycotoxins assessed (14) in this study using liquid chromatography-tandem mass spectrometry (LC-MS/MS) methodology for individual oat kernel analysis includes DON, DON-3G, 15-ADON, DAS, 15-AS, 3-ADON, ZEN, HT-2, T-2, T-2 triol, T-2 tetraol, OTA, OTB, and OTα.

## 2. Results and Discussion

### 2.1. Level of Mycotoxins in Whole Samples

The two oat batch samples studied contained 5 of the 14 analysed mycotoxins. The results of this analysis are summarised in Table 1, which shows the mean and the standard deviation of these mycotoxins in the oat samples assayed. The results showed that DON was present in significant concentrations in both samples, with mean levels of 3184.1 µg/kg in sample 1 and 3812.6 µg/kg in sample 2, resulting in a combined mean concentration of 3498.4 µg/kg. These values were above the EU legal limit for DON in oat batches (1750 µg/kg) [18], so the batch from which the sample originated would have to be rejected by the food industry. Its metabolite, DON-3G, was also detected at 851.0 µg/kg (sample 1) and 851.8 µg/kg (sample 2), with a combined mean of 851.4 µg/kg. 15-ADON was found at lower levels, with a combined mean concentration of 105.2 µg/kg across the two samples. Other notable findings include 3-ADON, which was detected at mean levels of 268.2 µg/kg in sample 1 and 288.4 µg/kg in sample 2, with a combined mean of 278.3 µg/kg. The detection of DON, DON-3G, 15-ADON, and 3-ADON confirms the importance of not relying solely on DON to assess human risk, as recommended by the European Commission [18]. ZEN was detected in both samples at relatively low levels, with a mean concentration of 52.2 µg/kg (below the EU legal limit for ZEN in oat batches [16]). No detectable levels were observed for DAS, 15-AS, HT-2, T-2, T-2 triol, T-2 tetraol, OTA, OTB, and OTα in the samples.

In line with our findings in Swedish oat samples specifically selected for high DON levels, other studies have reported high concentrations of DON, its derivatives, and ZEN in oats from Sweden, Finland, Poland, Norway, and Spain [5,25,26,27]. For example, organic and conventional oats harvested in Norway exhibited DON levels of 114 µg/kg and 426 µg/kg, respectively [26]. In Poland, an analysis of 22 oat samples indicated contamination rates of 81%, 38%, and 10% for DON, 3-ADON, and 15-ADON, respectively. The mean concentrations observed were 8.6 µg/kg for DON, 2.5 µg/kg for 3-ADON, and 0.2 µg/kg for 15-ADON [27]. In Sweden, 93 oat samples collected over two years (2010–2011) were screened for *Fusarium* toxins, revealing DON in over 90% of samples, with five samples exceeding the EU legal limit. 3-ADON was detected in 47% of samples with a maximum concentration of 1006 µg/kg, and ZEN was found in 22.5% of samples, while 15-ADON was not detected [5]. A nationwide survey in Finland of oats harvested in 2013 revealed high DON concentrations (mean 2690 µg/kg), with 32% of samples exceeding the EU legal limit. Elevated levels of 3-ADON were present in 55% of these samples, particularly in those with high DON concentrations that also had the highest levels of DON-3G reaching 6600 µg/kg (mean 806 µg/kg). ZEN was also found in 42% of the samples with a maximum level of 675 µg/kg [25]. In Spain, Tarazona et al. [28] found that the most prevalent mycotoxins in oat samples were ZEN (66%, mean 39.1 µg/kg), HT-2 (47%, mean 37.1 µg/kg), DON (34%, mean 81.4 µg/kg), fumonisin B1 (29%, mean 157.5 µg/kg), and T-2 (24%, mean 49.9 µg/kg) in years 2015–2019.

In our study, 3-ADON was detected more frequently and at higher concentrations than 15-ADON, which aligns with previous findings indicating that the 3-ADON chemotype is more common in oats than the 15-ADON chemotype and tends to dominate in Northern Europe [5,25] and in Spain [28]. Furthermore, 3-ADON levels showed a strong correlation with DON concentrations in Finnish oats [29].

In contrast, studies from the United Kingdom, Ireland, and Switzerland reported lower concentrations of DON, its derivatives, and ZEN in oat samples but elevated levels of T-2 and HT-2 [6,13,30]. This suggests that environmental conditions favouring T-2/HT-2-producing *Fusarium* species differ from those required by *F. culmorum* and *F. graminearum*, the primary producers of DON, its derivatives, and ZEN [6].

### 2.2. Comprehensive Analysis of Mycotoxins in Individual Oat Kernels

The analysis of mycotoxins in 200 individual oat kernels from two highly DON-contaminated samples is shown in Table 2. As far as we know, this is the first study about mycotoxins in individual oat kernels. It revealed significant variability in the occurrence and levels of contamination across the kernels. Other authors have observed this variability in wheat and maize [20,21,22]. As expected, DON was the most frequently detected mycotoxin, present in 64% of kernels in sample 1 and 76% of kernels in sample 2, with an overall occurrence of 70%. For sample 1, the average contamination considering all the kernels was 2160.3 µg/kg, and the average for the kernels exceeding the limit of detection (LOD) was 3338.6 µg/kg. For sample 2, the values were 5424.5 and 7115.2 µg/kg, respectively, indicating substantial variation between the two samples. The overall average contamination considering both samples was 3792.4 µg/kg, close to the value obtained from batch samples analysis (3498.4 µg/kg) (Table 1). The overall median DON contamination was 544.9 µg/kg across both samples, with individual kernels showing contamination levels ranging from below the LOD up to 296,741 µg/kg. As observed, some grains may contain extremely high levels of DON, potentially contaminating the rest of the sample once ground for analysis.

DON-3G, a modified form of DON, was also frequently detected. It occurred in 23% of kernels from sample 1 and 49% from sample 2, with a combined average and median concentration of 1488.5 µg/kg and 6.4 µg/kg, respectively. However, the maximum detected concentration of DON-3G reached 93,315 µg/kg in some kernels, suggesting high variability in contamination across individual grains.

Among other trichothecenes, 15-ADON was found in 4% and 8% of kernels in samples 1 and 2, respectively, with an overall average concentration of 97.3 µg/kg and an overall median concentration of 3.7 µg/kg. 3-ADON was detected in 20% of kernels across both samples, with a median concentration of 5.2 µg/kg and a maximum of 25,675 µg/kg, highlighting its sporadic but occasionally significant presence.

ZEN was detected in 1% of the kernels in sample 1 and 10% in sample 2, with an overall average concentration of 7.6 µg/kg. Although the median ZEN contamination was relatively low (2.1 µg/kg), some kernels showed contamination levels as high as 156.7 µg/kg.

Other mycotoxins, such as HT-2, T-2, DAS, and OTA, were detected in less than 5% of the kernels and at relatively low concentrations. For instance, HT-2 occurred in only 0.5% of kernels and T-2 in just 1%. T-2 derivatives, such as T-2 tetraol, were detected infrequently and in trace amounts. These results were anticipated, as trichothecenes A and OTA are produced by different fungal species and under distinct environmental conditions compared to trichothecenes B [6,13,30].

The data from individual kernels indicate considerable heterogeneity in mycotoxin contamination, but with DON and its derivatives dominating the profile. This heterogeneity may have significant implications for food safety and quality control, as bulk sample measurements may underestimate the risk posed by highly contaminated individual kernels.

### 2.3. Correlations, Co-Occurrence, and Ratios of Mycotoxins in Individual Oat Kernels

The analysis of individual oat kernels for mycotoxins provided insights into the correlations, co-occurrence patterns, and ratios among various mycotoxins. This information is crucial for understanding potential contamination patterns and relationships among mycotoxins within the kernels. Mycotoxin contamination in individual oat kernels has never been studied; therefore, these results are compared to the mycotoxin contamination data from oat samples.

**Figure 1:**
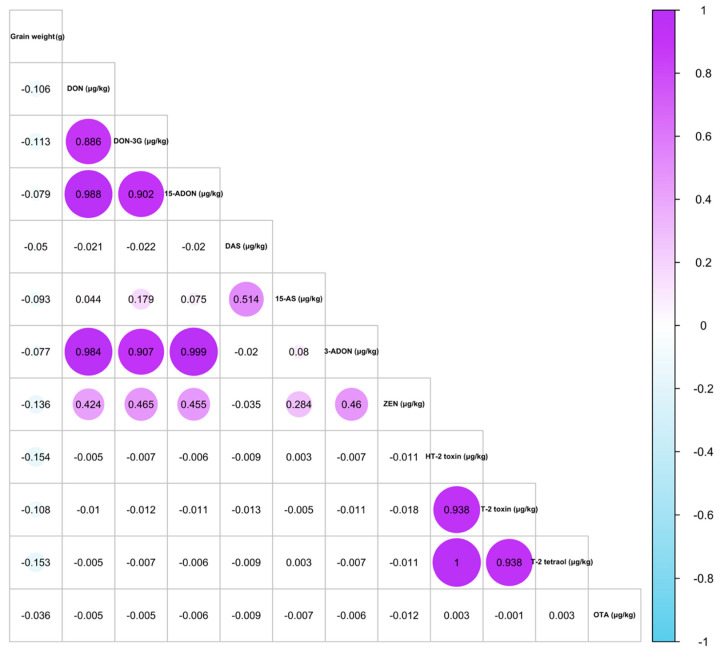
Correlation heat map matrix (represented with correlation coefficients) of the 14 analysed mycotoxins (µg/kg) and grain weight (g) in individual oat kernels. DON = deoxynivalenol; DON-3G = deoxynivalenol-3-glucoside; 15-ADON = 15-acetyldeoxynivalenol; DAS = diacetoxyscirpenol; 15-AS = 15-acetoxyscirpenol; 3-ADON = 15-acetyldeoxynivalenol; ZEN = zearalenone; OTA = ochratoxin A.

The correlation analysis (Figure 1) reveals significant relationships between certain mycotoxins. The most substantial correlations are observed among type B trichothecenes, particularly between DON and its modified forms. Specifically, DON and DON-3G exhibit a strong positive correlation (r = 0.886), as do DON and 15-ADON (r = 0.988) and DON and 3-ADON (r = 0.984). These high correlation values can be linked with these compounds (DON, 15-ADON, and 3-ADON) sharing similar biosynthetic pathways or environmental factors that favour their simultaneous production [5,25]. Moreover, DON and its modified forms are produced by the same species, such as *F. culmorum* and *F. graminearum* [6]. Such strong correlations are indicative of a co-occurrence trend within the trichothecene group, which may help identify kernels with higher contamination risk based on the presence of one compound as a proxy for others. Although no correlation of these mycotoxins in oat samples or individual oat kernels has been reported by other authors, some studies offer relevant insights. Tarazona et al. [28] observed that 3-ADON consistently co-occurs with DON. Similarly, Nathanail et al. [25] reported that oat samples with the highest levels of DON also exhibited the highest concentrations of DON-3G and 3-ADON. Furthermore, an analysis of Polish oat samples indicated that 3-ADON and 15-ADON were usually found in samples that also contained DON [27]. Notably, Hietaniemi et al. identified a strong correlation (not r value given) between 3-ADON and DON concentrations in oat samples [29].

In addition to the trichothecenes, moderate correlations are seen between DON and ZEN (r = 0.424) and between DON-3G and ZEN (r = 0.465). While these correlations were weaker than those within the trichothecenes, they suggested that ZEN co-occurs with DON and its modified forms in certain kernels. This is because DON, its derivatives, and ZEN are primarily produced by the same fungi (*F. graminearum* and *F. culmorum*), although under different environmental conditions [6]. Fredlund et al. [5] also found a similar correlation (r = 0.52) between DON and ZEN in oat samples. However, other mycotoxins, such as DAS and the type A trichothecenes (HT-2, T-2, T-2 tetraol), showed little to no significant correlation with DON or its related compounds, indicating that they may originate from distinct contamination events, species of *Fusarium*, or environmental conditions that differ from those of the type B trichothecenes [31]. However, type A trichothecenes showed a strong correlation, suggesting they frequently co-occur in the same kernels as observed by previous authors in oat samples [5,6,13,28,30]. Moreover, DAS and 15-AS had a moderate correlation (r = 0.514), suggesting a potential relationship between their occurrences.

A slight negative correlation is observed between kernel weight and mycotoxin levels, suggesting that higher contamination may be associated with lighter kernels, potentially due to fungal infection impacting kernel density and development. More detailed discussion about the influence of kernel weight on mycotoxin levels is explained in Section 2.4.

**Figure 2:**
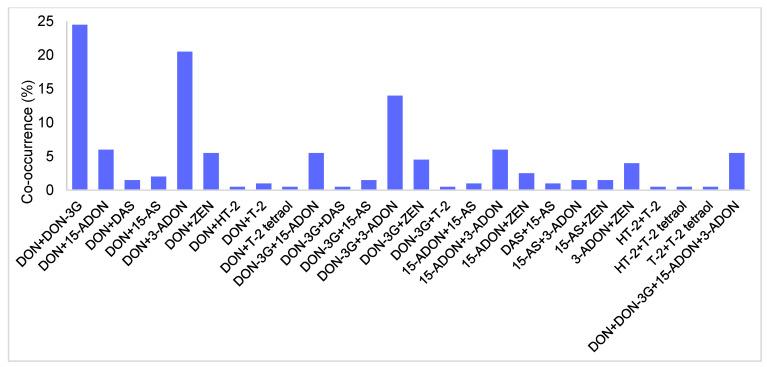
Co-occurrence (%) of the mycotoxins detected in individual oat kernels. Other co-occurrences were not found and, therefore, are not presented in this figure. DON = deoxynivalenol; DON-3G = deoxynivalenol-3-glucoside; 15-ADON = 15-acetyldeoxynivalenol; DAS = diacetoxyscirpenol; 15-AS = 15-acetoxyscirpenol; 3-ADON = 15-acetyldeoxynivalenol; ZEN = zearalenone; HT-2 = HT-2 toxin; T-2 = T-2 toxin.

The co-occurrence analysis (Figure 2) further supports these correlation findings. The most frequent co-occurrence is observed between DON and DON-3G, present in approximately 25% of contaminated kernels. Other frequent combinations include DON with 3-ADON (20%) and DON with 15-ADON (5%), which are observed at lower but still notable frequencies. Perkowski et al. [27] observed similar co-occurrence values in oat Polish samples, with 10% of the kernels with DON and 15-ADON and 33% with DON and 3-ADON. DON and ZEN co-occurred in 5% of the studied kernels. Interestingly, co-occurrences involving type A trichothecenes (HT-2, T-2, and T-2 tetraol) are rare or non-existent, supporting the idea that type A and type B trichothecenes have different contamination profiles in oat kernels. This separation of contamination profiles is crucial for risk assessment, as it suggests that controlling one group may not necessarily mitigate the presence of the other. As the European Commission has highlighted the importance of considering not just the content of DON but the sum of DON, 3-ADON, 15-ADON, and DON-3G to assess the human health risk [18], their co-occurrence was calculated. This co-occurrence was observed in approximately 5% of the kernels, which were typically the most highly contaminated with DON. These observed co-occurrences are since type B trichothecenes and ZEN are primarily produced by *F. culmorum* and *F. graminearum*, while type A trichothecenes are produced by *F. langsethiae* and *F. poae* [6].

Finally, the analysis of ratios when co-occurrences appeared (Table 3) provides additional insights into mycotoxin interactions within individual kernels. For example, the mean ratio of DON to DON-3G is approximately 13.0, with a median of 6.5 and a wide range from 0.5 to 135.1. This variability in ratios highlights the inconsistent conversion of DON to DON-3G across kernels, which could be influenced by factors such as variations in *Fusarium* infection on the plant, spikelets, and kernels, or environmental conditions within the same field [22,32]. Similar values and tendencies were observed with the ratios DON/15-ADON (mean = 27.5) and DON/3-ADON (mean = 17.2). Additionally, the ratio of DON to ZEN was notably high (mean 257.2), further indicating that while these toxins may co-occur, their relative concentrations vary significantly, possibly due to differing metabolic pathways. The HT-2/T-2 ratio was 2.3, aligning with other studies that report HT-2 toxin concentrations are typically higher than T-2 levels in European raw oats, with an average ratio of 1.9 [33].

In summary, the correlation, co-occurrence, and ratios analyses suggest that DON and its modified forms are frequently found together in individual oat kernels, whereas type A trichothecenes and other unrelated mycotoxins, such as DAS and OTA, appear to occur independently. Moreover, the ratios between mycotoxins varied widely, suggesting that while they often co-occur, the relative concentrations can differ significantly between individual kernels. These findings underscore the complex contamination profiles in individual oat kernels.

### 2.4. Correlation of Kernel Weight with Mycotoxin Levels

**Figure 3:**
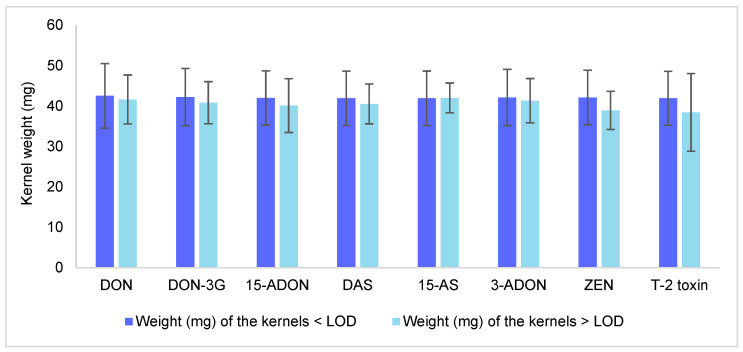
Kernel weight (mg) according to the contamination of the detected mycotoxins (above the LOD or below the LOD). LOD = limit of detection; DON = deoxynivalenol; DON-3G = deoxynivalenol-3-glucoside; 15-ADON = 15-acetyldeoxynivalenol; DAS = diacetoxyscirpenol; 15-AS = 15-acetoxyscirpenol; 3-ADON = 15-acetyldeoxynivalenol; ZEN = zearalenone.

The correlation of mycotoxin contamination with the weight of individual oat kernels was evaluated, as shown in Figure 3. While no significant differences (*p* > 0.05) were observed between the weight of contaminated and uncontaminated kernels for the mycotoxins analysed, a general trend was noted: contaminated kernels (>LOD) tended to have slightly lower weights compared to uncontaminated kernels (<LOD) (average reduction of 3.9%). This outcome agreed with the one observed in Figure 1 (slight negative correlation between mycotoxins and kernel weight). This trend was visible across most of the mycotoxins, including DON, DON-3G, 15-ADON, 3-ADON, DAS, and ZEN. Despite the lack of statistical significance, the observed trend suggests that *Fusarium* infection and its subsequent production of mycotoxins may reduce kernel weight to some extent.

Martinelli et al. [34] reported an average weight reduction of 3.2% in kernels naturally infected by *F. graminearum*. In contrast, Yan et al. [11] investigated which oat traits might correlate with high DON content and found that cultivars with higher DON contamination tended to have increased protein content and a greater hull-to-groat ratio, though the correlation with kernel weight was weak. Martin et al. [35] studied the effects of *F. langsethiae* infection on protein, β-glucan, and grain weight in oats, observing that while the fungus affected β-glucan levels, it had no significant impact on kernel weight or protein content.

For OTA, HT-2, and T-2 tetraol, contamination was found in one kernel each, limiting the ability to draw any meaningful conclusions for these specific toxins.

### 2.5. Potential Impact of Observed Results in Sampling Outcomes, Food Safety, and Mycotoxin Detection

**Figure 4:**
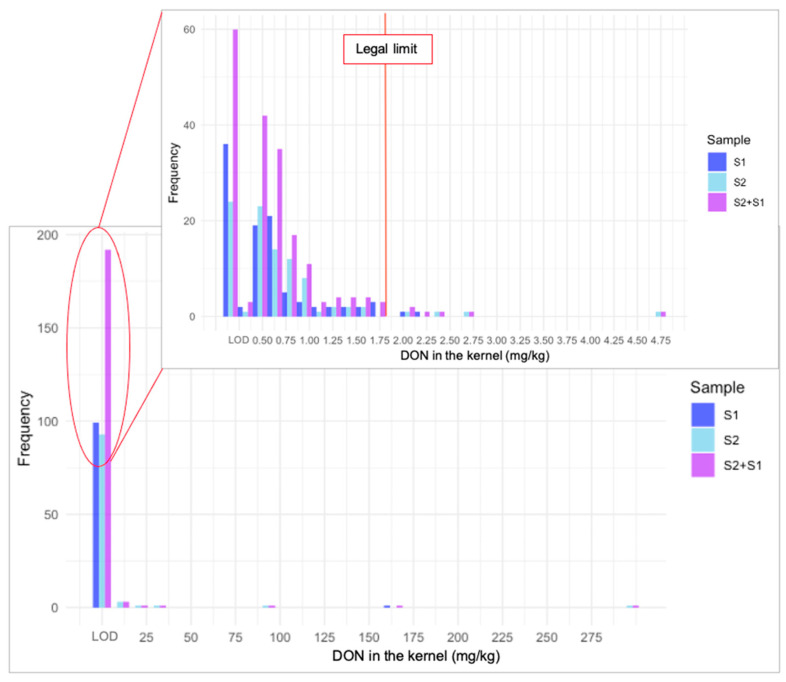
Distribution of deoxynivalenol (DON) content (mg/kg) in kernels from sample 1, sample 2, and combined samples, with an expanded view near the legal limit for DON (1.75 mg/kg).

Out of the 200 oat kernels analysed, 16 would exceed the legal limit for DON (1750 µg/kg), 4 would exceed the threshold for ZEN (100 µg/kg), and only 1 would exceed the limit for the combined T-2 and HT-2 (1250 µg/kg). Regarding DON, the results revealed a broad distribution, consistent with a log-normal distribution, characterised by a large number of kernels with undetected or low DON levels and a few with exceptionally high concentrations (Figure 4). This pattern suggests that the overall contamination of the batch is heavily influenced by these highly contaminated kernels. Similar trends were observed for ZEN (Figure S1), DON-3G (Figure S2), 15-ADON (Figure S3), 3-ADON (Figure S4), DAS (Figure S5), 15-AS (Figure S6), and the sum of T-2 and HT-2 (Figure S7). Femenias et al. [36] observed a similar heterogeneous distribution with DON in individual wheat kernels, but mycotoxin distribution in single kernels has never been studied in oats.

Table 4 presents the probabilities of exceeding the legal limits based on the detected levels of DON, ZEN, and the sum of T-2 and HT-2, as evaluated through both empirical and theoretical approaches. These probabilities were calculated across varying selected kernel percentages, ranging from 0.5% to 100% of the dataset, with 100 iterations (trying to simulate the number of elemental samples based on the EU, although this study is using individual oat kernels) and according to the legal thresholds established by EU regulations [16,17,18,19].

The DON content in the oat batch samples exceeded the legal limit set by the EU (Table 1); it could be assumed that the batch would need to be rejected for the food industry, although this conclusion is based on the analysis of only two samples (40 g each). The results showed that rejection probabilities increased as the selected kernels percentage rose, with full rejection (100%) observed when the entire dataset was analysed (200 kernels = 100% selected kernels). At smaller selected kernel percentages, the probabilities of acceptance and rejection varied due to the inherent variability within the dataset. For instance, 10% selected kernels resulted in approximately 55% rejection probability, although the sample had an average contamination higher than 3000 µg/kg of DON. The empirical probabilities generally indicated a higher likelihood of oat sample batch acceptance compared to the theoretical probabilities derived from the log-normal distribution. This discrepancy underscores the potential influence of distributional assumptions on theoretical evaluations. For instance, although working with whole samples, Tittlemier et al. [21] suggested that current sampling plans for wheat and maize can introduce high variance in mycotoxin testing and risk of misclassifying consignments.

The oat batch samples accomplished with ZEN EU legal limits (Table 1). Therefore, the food industry would accept that batch according to ZEN content. The ZEN results showed that for empirical and theoretical methods, the acceptance probabilities were almost 100% in all the studied selected kernel percentages, indicating the low possibility of exceeding the legal limit for ZEN.

There was an undetectable level of T-2 and HT-2 in the oat batch samples (Table 1). In the sampling statistics study, empirical probabilities consistently indicated legal limit compliance across all selected kernel percentages. This finding can be attributed to the limited contamination observed in the dataset, where only two individual kernels were identified as presenting values higher than the LOD for these toxins. Consequently, theoretical probabilities could not be reliably calculated for this group due to the insufficient data points available to fit a log-normal distribution.

The European Commission [19] established that for cereals, a sample of 10 kg should be collected to analyse mycotoxins in batches ranging from 100,000 to 1,500,000 kg, which represents between 0.1% and 0.0007% of the total batch. Although the study was conducted differently, because it was focused on individual oat kernels and not on samples, we can anticipate some potential situations related to the sampling methodology. In this study, the minimum percentage analysed was 0.5%, corresponding to 1 of 200 kernels. At this minimum sampling percentage (0.5%), it was highly probable (84–93%) that an oat batch sample containing more than 3000 µg/kg of DON would be accepted, posing a significant risk. If a lower percentage had been used, as required by EU legislation, the probability of accepting a batch with high DON contamination would have been even greater. In the case of ZEN or HT-2 and T-2, the batches should be accepted, as also shown by the probabilities in Table 4. Therefore, in this study, the problem lies in accepting sample batches with high mycotoxin content rather than rejecting those we should accept. This is due to the way mycotoxins are distributed in the individual oat kernels, where only a few kernels have very high contamination, which raises the average contamination of the sample. The measurement uncertainty of the method directly influences the likelihood of false-positive or false-negative results, which in turn impacts the decision-making process for accepting or rejecting a lot. By incorporating measurement uncertainty into the decision rule, the process ensures a more robust evaluation that minimises the risk of incorrect conclusions and enhances the reliability of the outcomes.

Overall, the findings emphasise the critical role of mycotoxin heterogeneity in accurately determining batch compliance with legal limits, especially for batches that need to be rejected. A smaller percentage of analysed kernels may underestimate the true mycotoxin levels, leading to higher probabilities of acceptance and potentially compromising food safety. This has significant implications for the enforcement of EU regulations aimed at minimising consumer exposure to harmful mycotoxins and ensuring the safety of oat-based products in the food supply chain.

## 3. Conclusions

The high levels of DON contamination in oat sample batches were found to depend on less than 10% of the kernels. These contaminated kernels, which exceed the legal limit, are often extremely contaminated, with one kernel reaching a maximum concentration of 296,741 µg/kg. Such severe contamination drives the overall sample batch to exceed the legal limit (3498 µg/kg), which would lead to batch rejection. Furthermore, these highly contaminated kernels also contain modified forms of DON (DON-3G, 15-ADON, and 3-ADON) and may contain ZEN, as suggested by the correlation and co-occurrence data. This poses an increased food safety concern, as routine analysis for DON and ZEN typically excludes DON-modified forms, which are therefore not accounted for in exposure assessments.

Interestingly, the majority (90%) of the kernels were free from the studied mycotoxins. However, some grains that lacked DON showed the presence of other mycotoxins, such as type A trichothecenes or *Aspergillus*-related toxins, suggesting that these mycotoxins are produced by different fungal species or under distinct environmental conditions.

These findings highlight the need for targeted strategies to remove highly contaminated grains from oat batches. Such techniques could prevent the rejection of entire batches, contributing to both sustainability and economic efficiency within the food industry.

Additionally, the study highlights the significant heterogeneity in mycotoxin contamination within oat sample batches, stressing the crucial need for adequate sample sizes (percentage of selected kernels in our study) and enhanced sampling methodologies to minimise the risk of misclassifying oat batches. Improving detection accuracy and ensuring compliance with regulatory limits will ultimately contribute to the production of safer oat-based food products, thereby protecting consumer health and building trust.

## 4. Materials and Methods

### 4.1. Samples

Two samples of white oat (*Avena sativa* L.) were selected according to their high DON content. These were collected in the Västra Götaland region (Sweden) during the period 2021–2022.

### 4.2. Chemicals and Reagents

All mycotoxin standards were supplied by Romer Lab (Tulln, Austria) and were dissolved in acetonitrile. These include DON (100 µg/mL), DON-3G (50.1 µg/mL), 15-ADON (100.2 µg/mL), DAS (100.1 µg/mL), 15-AS (52.2 µg/mL), 3-ADON (100 µg/mL), ZEN (100 µg/mL), HT-2 (100 µg/mL), T-2 (100 µg/mL), T-2 triol (50.5 µg/mL), T-2 tetraol (50.3 µg/mL), OTA (10.1 µg/mL), OTB (10 µg/mL), and OTα (10.2 µg/mL).

Ultrapure water was obtained with PURELAB^®^ Chorus 1 (ELGA LabWater; Buckinghamshire, UK). Ammonium acetate (>99%, analytical reagent grade), acetic acid glacial (99.7%, HPLC grade), acetonitrile (LC-MS grade), and methanol (LC-MS grade) were obtained from Fisher Scientific (Waltham, MA, USA).

### 4.3. Preparation of Standard Solutions

A mixed stock standard solution of DON, DON-3G, 15-ADON, DAS, 15-AS, 3-ADON, ZEN, HT-2, T-2, T-2 triol, T-2 tetraol, OTA, OTB, and OTα was prepared in acetonitrile and stored at −20 °C in a sealed vial. A calibration curve was prepared by appropriate dilution of the standard solutions following a matrix-matched calibration procedure to be analysed afterwards by LC-MS/MS.

### 4.4. Sample Preparation

The mycotoxins (DON, DON-3G, 15-ADON, DAS, 15-AS, 3-ADON, ZEN, HT-2, T-2, T-2 triol, T-2 tetraol, OTA, OTB, and OTα) from 200 oat kernels, 100 from each sample, were individually extracted. Oat kernels were ground using the Precellys^®^ homogeniser (Bertin Technologies, Yvelines, France). Each oat kernel was incorporated in an Eppendorf tube with one 7 mm stainless steel bead and homogenised at a speed of 5200 rpm for 2 cycles of 10 s with a 5-s pause in between. Subsequently, 260 μL of extraction solvent, consisting of acetonitrile, ultrapure water, and acetic acid in a ratio of 79:20:1 (*v/v/v*), was added to each tube. The tubes were then transferred to a shaker plate and placed on a rotary shaker at 600 rpm and 25 °C for 90 min (miniShaker VWR; VWR, Leighton Buzzard, UK). After shaking, the extracts were centrifuged for 5 min at 13,000 rpm (Centrifuge 5417S Eppendorf, Stevenage, UK) at 24 °C. The supernatants (75 μL) were mixed with 75 μL of dilution solvent, which contains acetonitrile, ultrapure water, and acetic acid in a ratio of 20:79:1 (*v/v/v*), and transferred to vials with 250 μL micro inserts.

Moreover, the same mycotoxins were analysed for each oat sample (*n* = 5). Forty g of each sample were ground with an IKA^®^ A11 Basic mill (Darmstadt, Germany). After mixing carefully, 3 g were mixed with 15 mL of the same extraction solvent in 50 mL Falcon^®^ tubes, followed by shaking at 1200 rpm and 25 °C for 90 min (Heidolph™ Multi Rear; Fisher Scientific, Waltham, MA, USA). After shaking, the extracts were centrifuged for 5 min at 10,000 rpm (Multifuge X Pro Series; Thermo Fisher Scientific, Waltham, MA, USA) at 22 °C. The supernatants (750 μL) were mixed with 750 μL of the previous dilution solvent and transferred to vials.

All vials were stored in a freezer at −20 °C until analysis by LC-MS/MS.

### 4.5. LC-MS/MS Parameters

Analysis by LC-MS/MS was performed with an Exion series LC system coupled to a 6500+ hybrid triple quadrupole-linear ion trap mass spectrometer (qTRAP-MS) system also coupled with IonDrive Turbo Spray (both Sciex Technologies, Warrington, UK). Chromatographic separation was achieved on a reversed-phase ACE-3 C18 column (2.1× 100 mm, 3 μm particle size; Hichrom, Berkshire, UK) equipped with an ACE-3 guard cartridge maintained at 60 °C. The gradient elution was carried out with solvent A, which contained ultrapure water, methanol, and acetic acid in a ratio of 89:10:1 (*v/v/v*) and solvent B, which contained ultrapure water, methanol, and acetic acid in a ratio of 2:97:1 (*v/v/v*), both supplemented with 5 mM ammonium acetate to promote the formation of ammonium adducts. The applied gradient was 20 min long as described: 0 min, 0% B; 2 min, 0% B; 5 min, 50% B; 14 min, 100% B; 18 min, 100% B; 19 min, 0% B. Flow rate was set at 0.6 mL/min, and the injection volume was 1 μL.

The MS analysis operated in positive (ESI+) and negative (ESI−) electrospray ionisation (ESI) modes depending on the mycotoxin studied. The source conditions were set as follows: curtain gas 40 psi, collision gas medium, ion spray voltage −4500 V (in negative) and 5500 V (in positive), temperature 400 °C, ion source gas 1 60 psi, and ion source gas 2 60 psi. Multiple reaction monitoring (MRM) acquisition mode was applied. The optimised retention time, precursor ion, and product ions obtained via direct infusion (syringe method) of reference standard solutions for the studied mycotoxins are reported in Table 5. The MS/MS parameters (collision energy, declustering potential, entrance potential, and collision cell exit potential), presented in Table 5, were also optimised by infusion on each compound separately and checked on the mixed stock standard solution. Two or three m/z transitions were monitored for each mycotoxin. The analyte identification was realised based on the assessment of retention time and the quantifier and qualifier transitions. Data acquisition was conducted with Analyst^®^ version 1.6.3 and quantification through MultiQuant™ version 3.0.3.

### 4.6. Method Validation

The analytical method for quantifying DON, DON-3G, 15-ADON, DAS, 15-AS, 3-ADON, ZEN, HT-2, T-2, T-2 triol, T-2 tetraol, OTA, OTB, and OTα in individual oat kernels and oat samples by LC-MS/MS was validated. Selectivity was checked by injecting 1 µL of standard solution at least three times, comparing retention time and peak resolution between injections. Selectivity was ensured by analysing blank, matrix, and spiked samples to check for co-eluting peaks or signals at the same *m/z* transitions. For the linearity check, a matrix-matched calibration curve of nine concentration levels for each mycotoxin was injected into the system. A coefficient of determination (R^2^) ≥ 0.98 was chosen as the acceptability criterion. Likewise, the precision was evaluated by spiking blank individual oat kernels at the level according to Table 6 on three different days (*n* = 3, 3, 10), and the recoveries and the relative standard deviation (RSD) were calculated for each mycotoxin (Table 6). Method performance was assessed according to Commission Regulation (EC) No. 2023/2782 [19]. OTB and OTα recoveries and RSDs could not be calculated at the set spiking level. Moreover, the guidance document on the estimation of LOD and limit of quantification (LOQ) for measurements in the field of contaminants in feed and food [37] was followed (Table 6). The estimation of the LOD was performed using pseudo-blank samples for all toxins, except for OTB and OTα, where the limits were estimated through standard calibration.

### 4.7. Statistical Analysis

The statistical analyses and graphical displays were performed using RStudio version 4.2.3 (Posit, Boston, MA, USA) and Microsoft Excel version 16.87 (Microsoft, Redmond, Washington, DC, USA). To calculate the overall average and median contamination for each mycotoxin, a value of LOD/√2 was used for kernels with values below the LOD [38]. To assess the probability that the average levels of DON, ZEN, and the sum of T-2 and HT-2 in oat samples selecting different percentages of kernels exceeded the EU thresholds (1750 µg/kg, 100 µg/kg, and 1250 µg/kg, respectively), both theoretical and empirical approaches were employed. Firstly, to calculate the theoretical probability of accepting or rejecting the oat batch samples, the distribution of the dataset—consisting of toxin levels measured in individual oat kernels—was analysed. Secondly, the log-normal distribution was the best fit according to QQ plots, cumulative distribution function plots, and PP plots. Subsequently, randomly selected kernels (from the 200 kernels analysed) ranging from 0.5% to 100% of the total dataset were drawn with replacements to calculate theoretical and empirical probabilities. For each percentage of selected kernels, 100 iterations were conducted (as the subsampling established for cereal batches by the EU regulations [19]), and the mean toxin level was calculated for each iteration. The probability of exceeding the thresholds was determined by the proportion of iterations where the mean surpassed the specified limit. The empirical method provided a distribution-free estimation and used the original dataset, contrasting with the theoretical approach based on the log-normal assumption of the data.

## Figures and Tables

**Table 1:** Overview of the content of the 14 analysed mycotoxins (µg/kg) in the oat batch samples. SD = standard deviation; N. d. = not detected; DON = deoxynivalenol; DON-3G = deoxynivalenol-3-glucoside; 15-ADON = 15-acetyldeoxynivalenol; DAS = diacetoxyscirpenol; 15-AS = 15-acetoxyscirpenol; 3-ADON = 15-acetyldeoxynivalenol; ZEN = zearalenone; OTA = ochratoxin A; OTB = ochratoxin B; OT*α* = ochratoxin-alpha.

Mycotoxin	Sample 1		Sample 2		Sample 1 + Sample 2	
	Mean Concentration, µg/kg	SD, µg/kg	Mean Concentration, µg/kg	SD, µg/kg	Mean Concentration, µg/kg	SD, µg/kg
DON	3184	57.9	3813	97.6	3498	77.8
DON-3G	851.0	70.0	851.8	67.4	851.4	68.7
15-ADON	98.6	9.3	111.7	6.3	105.2	7.8
DAS	N. d.	N. d.	N. d.	N. d.	N. d.	N. d.
15-AS	N. d.	N. d.	N. d.	N. d.	N. d.	N. d.
3-ADON	268.2	12.7	288.4	19.5	278.3	16.1
ZEN	55.7	2.4	48.7	1.7	52.2	2.1
HT-2	N. d.	N. d.	N. d.	N. d.	N. d.	N. d.
T-2	N. d.	N. d.	N. d.	N. d.	N. d.	N. d.
T-2 triol	N. d.	N. d.	N. d.	N. d.	N. d.	N. d.
T-2 tetraol	N. d.	N. d.	N. d.	N. d.	N. d.	N. d.
OTA	N. d.	N. d.	N. d.	N. d.	N. d.	N. d.
OTB	N. d.	N. d.	N. d.	N. d.	N. d.	N. d.
OTα	N. d.	N. d.	N. d.	N. d.	N. d.	N. d.

**Table 2:** Overview of the 14 analysed mycotoxins in 200 individual oat kernels from two highly DON-contaminated samples.

Sample	Mycotoxin	Occurrence, %	Overall Average Contamination, µg/kg ^1^	Average Contamination (Kernels > LOD), µg/kg	Overall Median Contamination, µg/kg ^1^	Median Contamination (Kernels > LOD), µg/kg	Range (Min–Max), µg/kg
1	DON	64 (64%)	2160	3339	477.1	579.7	N. d.–164,124
	DON-3G	23 (23%)	771.9	3335	6.5	133.5	N. d.–68,938
	15-ADON	4 (4%)	52.8	1227	3.7	103.1	N. d.–4643
	DAS	2 (2%)	2.1	79.7	0.4	79.7	N. d.–82.5
	15-AS	2 (2%)	7.6	105.8	5.2	105.8	N. d.–137.6
	3-ADON	20 (20%)	161.0	784.4	5.1	68.6	N. d.–14,168
	ZEN	1 (1%)	3.6	145.0	2.0	145.0	N. d.–145.0
	HT-2	1 (1%)	25.1	1175	12.5	1175	N. d.–1174
	T-2	1 (1%)	7.5	514.2	2.2	514.2	N. d.–514.2
	T-2 triol	N. d.	N. d.	N. d.	N. d.	N. d.	N. d.
	T-2 tetraol	1 (1%)	26.8	1260	13.3	1260	N. d.–1260
	OTA	1 (1%)	0.6	22.04	0.3	22.04	N. d.–22.0
	OTB	N. d.	N. d.	N. d.	N. d.	N. d.	N. d.
	OTα	N. d.	N. d.	N. d.	N. d.	N. d.	N. d.
2	DON	76 (76%)	5425	7115	557.6	713.8	N. d.–296,741
	DON-3G	26 (26%)	2125	8154	6.7	205.1	N. d.–93,315
	15-ADON	8 (8%)	141.7	1724	3.7	763.7	N. d.–8856
	DAS	3 (3%)	2.4	63.1	0.45	60.0	N. d.–74.4
	15-AS	3 (3%)	8.3	82.6	5.27	76.9	N. d.–103.0
	3-ADON	21 (21%)	411.9	1941	5.35	140.6	N. d.–25,675
	ZEN	10 (10%)	11.6	95.0	2.06	91.8	N. d.–156.7
	HT-2	N. d.	N. d.	N. d.	N. d.	N. d.	N. d.
	T-2	1 (1%)	4.5	189.8	2.28	189.8	N. d.–189.8
	T-2 triol	N. d.	N. d.	N. d.	N. d.	N. d.	N. d.
	T-2 tetraol	N. d.	N. d.	N. d.	N. d.	N. d.	N. d.
	OTA	N. d.	N. d.	N. d.	N. d.	N. d.	N. d.
	OTB	N. d.	N. d.	N. d.	N. d.	N. d.	N. d.
	OTα	N. d.	N. d.	N. d.	N. d.	N. d.	N. d.
1 + 2	DON	140 (70%)	3792	5389	544.9	644.5	N. d.–296,741
	DON-3G	49 (24.5%)	1449	5892	6.64	160.8	N. d.–93,315
	15-ADON	12 (6%)	97.3	1558	3.74	231.6	N. d.–8856
	DAS	5 (2.5%)	2.2	69.7	0.45	74.4	N. d.–82.5
	15-AS	5 (2.5%)	8.0	91.9	5.31	76.9	N. d.–137.6
	3-ADON	41 (20.5%)	286.5	1377	5.24	74.9	N. d.–25,675
	ZEN	11 (5.5%)	7.6	99.5	2.05	93.5	N. d.–156.7
	HT-2	1 (0.5%)	19.7	1175	12.64	1175	N. d.–1174
	T-2	2 (1%)	6.0	352.0	2.27	352.0	N. d.–514.2
	T-2 triol	N. d.	N. d.	N. d.	N. d.	N. d.	N. d.
	T-2 tetraol	1 (0.5%)	21.0	1260	13.39	1260	N. d.–1260
	OTA	1 (0.5%)	0.5	22.0	0.35	22.0	N. d.–22.0
	OTB	N. d.	N. d.	N. d.	N. d.	N. d.	N. d.
	OTα	N. d.	N. d.	N. d.	N. d.	N. d.	N. d.

^1^ To calculate the overall average and median contamination for each mycotoxin, a value of LOD/√2 was used for kernels with values below the LOD. LOD = limit of detection; N. d. = not detected; DON = deoxynivalenol; DON-3G = deoxynivalenol-3-glucoside; 15-ADON = 15-acetyldeoxynivalenol; DAS = diacetoxyscirpenol; 15-AS = 15-acetoxyscirpenol; 3-ADON = 15-acetyldeoxynivalenol; ZEN = zearalenone; OTA = ochratoxin A; OTB = ochratoxin B; OT*α* = ochratoxin-alpha.

**Table 3:** Ratios between mycotoxin concentration (µg/kg) when co-occurrence existed. DON = deoxynivalenol; DON-3G = deoxynivalenol-3-glucoside; 15-ADON = 15-acetyldeoxynivalenol; DAS = diacetoxyscirpenol; 15-AS = 15-acetoxyscirpenol; 3-ADON = 15-acetyldeoxynivalenol; ZEN = zearalenone; HT-2 = HT-2 toxin; T-2 = T-2 toxin.

Ratios	Mean	Median	Range (Min–Max)
DON/DON-3G	13.0	6.5	0.5–135.1
DON/15-ADON	27.5	26.3	7.9–63.7
DON/DAS	10.8	10.9	8.6–12.9
DON/15-AS	206.2	194.7	5.0–430.4
DON/3-ADON	17.2	11.6	6.1–135.3
DON/ZEN	257.2	25.5	6.6–1894
DON/HT-2	0.6	0.6	0.6–0.6
DON/T-2	2.5	2.5	1.4–3.6
DON/T-2 tetraol	0.6	0.6	0.6–0.6
DON-3G/15-ADON	9.5	4.4	0.5–48.3
DON-3G/DAS	1.4	1.4	1.4–1.4
DON-3G/15-AS	328.7	55.9	6.1–924.1
DON-3G/3-ADON	8.1	1.9	0.1–59.2
DON-3G/ZEN	179.3	35.8	1.6–787.9
DON-3G/T-2	1.3	1.3	1.3–1.3
15-ADON/15-AS	18.6	18.6	18.1–19.1
15-ADON/3-ADON	0.5	0.4	0.3–1.2
15-ADON/ZEN	18.6	16.3	1.5–56.5
DAS/15-AS	0.6	0.6	0.6–0.6
15-AS/3-ADON	0.03	0.02	0.02–0.5
15-AS/ZEN	0.7	0.8	0.5–0.9
3-ADON/ZEN	35.1	4.0	0.9–163.9
HT-2/T-2	2.3	2.3	2.3–2.3
HT-2/T-2 tetraol	0.9	0.9	0.9–0.9
T-2/T-2 tetraol	0.4	0.4	0.4–0.4

**Table 4:** Empirical and theoretical probabilities (0–1) of accepting or rejecting oat batch samples based on EU thresholds for deoxynivalenol (DON) (1750 µg/kg), zearalenone (ZEN) (100 µg/kg), and T-2 + HT-2 toxins (1250 µg/kg) across the selection of different percentages of kernels (maximum number of kernels = 200). Underlined entries indicate whether the batch should be accepted or rejected based on the detected mycotoxin content in the samples.

Percentage of Selected Kernels	DON				ZEN				T-2+HT-2 Toxins	
	Empirical Probability		Theoretical Probability (Log-Normal)		Empirical Probability		Theoretical Probability (Log-Normal)		Empirical Probability	
	Accept	Reject	Accept	Reject	Accept	Reject	Accept	Reject	Accept	Reject
0.5%	0.93	0.07	0.84	0.16	0.97	0.03	1	7·10^−5^	1	0
1%	0.95	0.05	0.87	0.13	1	0	1	1·10^−4^	1	0
2%	0.84	0.16	0.75	0.25	1	0	1	5·10^−4^	1	0
3%	0.79	0.21	0.65	0.35	1	0	1	5·10^−4^	1	0
4%	0.75	0.25	0.62	0.38	1	0	1	6·10^−4^	1	0
5%	0.65	0.35	0.57	0.43	1	0	1	5·10^−4^	1	0
6%	0.54	0.36	0.53	0.47	1	0	1	2·10^−4^	1	0
7%	0.60	0.40	0.50	0.50	1	0	1	1·10^−4^	1	0
8%	0.58	0.42	0.49	0.51	1	0	1	8·10^−5^	1	0
9%	0.55	0.45	0.48	0.52	1	0	1	6·10^−5^	1	0
10%	0.55	0.45	0.48	0.52	1	0	1	4·10^−5^	1	0
15%	0.53	0.47	0.43	0.57	1	0	1	7·10^−8^	1	0
20%	0.53	0.47	0.39	0.61	1	0	1	2·10^−9^	1	0
25%	0.39	0.61	0.34	0.66	1	0	1	5·10^−11^	1	0
30%	0.39	0.61	0.34	0.66	1	0	1	2·10^−14^	1	0
40%	0.23	0.77	0.26	0.74	1	0	1	3·10^−14^	1	0
50%	0.21	0.79	0.20	0.80	1	0	1	0	1	0
75%	0.15	0.85	0.15	0.85	1	0	1	0	1	0
100%	0	1	0	1	1	0	1	0	1	0

**Table 5:** Optimised LC-MS/MS parameters for the analysis of mycotoxins in oats. DON = deoxynivalenol; DON-3G = deoxynivalenol-3-glucoside; 15-ADON = 15-acetyldeoxynivalenol; DAS = diacetoxyscirpenol; 15-AS = 15-acetoxyscirpenol; 3-ADON = 15-acetyldeoxynivalenol; ZEN = zearalenone; OTA = ochratoxin A; OTB = ochratoxin B; OTα = ochratoxin-alpha.

Mycotoxin	Retention Time (Min)	Precursor Ion (*m/z*)	Molecular Ion	Product Ions (*m/z*) ^1^	Collision Energy (V)	Declustering Potential (V)	Entrance Potential (V) ^2^	Collision Cell Exit Potential (V)
DON	1.77	297.0	[M+H]^+^	**249.1** 203.2	21	91	-	20
DON-3G	1.93	517.1	[M+CH_3_CO_2_]^−^	**457.2** 417.1	−20 −30	−115	−10	−19 −11
15-ADON	4.37	339.0	[M+H]^+^	**260.9** 320.8 137.0	15	56	-	14
					11			16
					15			22
DAS	4.33	384.2	[M+NH_4_]^+^	**307.0** 246.9	15 21	71	-	28
								14
15-AS	4.96	342.2	[M+NH_4_]^+^	**265.1** 307.2	13	71	-	26
								8
3-ADON	4.33	339.0	[M+H]^+^	**231.0** 203.0 43.1	15	31	-	26
					17			22
					75			20
ZEN	6.76	317.1	[M-H]^−^	**175.0** 131.2	−34 −42	−100	−10	−13
								−8
HT-2	6.06	442.0	[M+NH_4_]^+^	**262.9** 215.0	17	1	-	24
								12
T-2	6.58	484.0	[M+NH_4_]^+^	**305.0** 215.1	19 27	26	-	14
								12
T-2 triol	5.57	400.2	[M+NH_4_]^+^	**215.2** 281.3	17 13	71	-	12
								16
T-2 tetraol	1.02	316.2	[M+NH_4_]^+^	**215.2** 281.2	13	61	-	16
								8
OTA	6.77	404.0	[M+H]^+^	**239.0** 102.0	37 105	91	-	16
						102		14
OTB	6.06	370.1	[M+H]^+^	**205.0** 103.1	33 77	86	-	12
								16
OTα	4.65	254.9	[M-H]^−^	**210.9** 166.9	−24 −36	−90	−10	−11

^1^ Quantifier ion is indicated in bold. ^2^ The sign of the entrance potential indicates whether the instrument is operating in positive ion mode (positive potential) or negative ion mode (negative potential), reflecting the type of ions being analysed.

**Table 6:** Method performances for mycotoxin analysis in oats by optimised LC-MS/MS methodology. DON = deoxynivalenol; DON-3G = deoxynivalenol-3-glucoside; 15-ADON = 15-acetyldeoxynivalenol; DAS = diacetoxyscirpenol; 15-AS = 15-acetoxyscirpenol; 3-ADON = 15-acetyldeoxynivalenol; ZEN = zearalenone; OTA = ochratoxin A; OTB = ochratoxin B; OTα = ochratoxin-alpha.

Mycotoxin	Spiking Level, µg/kg	LOD, µg/L	LOQ, µg/L	Recovery ± SD, % (*n* = 16)	RSD, % (*n* = 3, 3, 10)		Linearity, R^2^
DON	1750	5.0	16.5	100.4 ± 8.9	Day 1 = 10.6	8.8	0.992
					Day 2 = 3.6		
					Day 3 = 9.8		
DON-3G	100	0.4	1.4	84.2 ± 9.1	Day 1 = 6.9	10.8	0.992
					Day 2 = 7.2		
					Day 3 = 12.4		
15-ADON	75	0.4	1.2	115.3 ± 5.2	Day 1 = 0.8	4.5	0.994
					Day 2 = 2.3		
					Day 3 = 5.6		
DAS	75	0.05	0.2	124.0 ± 3.7	Day 1 = 3.1	3.0	0.990
					Day 2 = 1.5		
					Day 3 = 3.3		
15-AS	75	0.5	1.6	111.0 ± 8.5	Day 1 = 7.5	7.7	0.987
					Day 2 = 10.6		
					Day 3 = 7.3		
3-ADON	75	0.5	1.5	115.1 ± 5.8	Day 1 = 1.2	5.1	0.990
					Day 2 = 1.9		
					Day 3 = 6.2		
ZEN	100	0.2	0.7	115.8 ± 5.7	Day 1 = 4.4	4.9	0.995
					Day 2 = 0.3		
					Day 3 = 5.0		
HT-2 toxin	750	1.2	4.0	114.6 ± 6.5	Day 1 = 3.8	5.6	0.982
					Day 2 = 5.1		
					Day 3 = 6.4		
T-2 toxin	250	0.2	0.8	119.2 ± 3.7	Day 1 = 1.5	3.1	0.981
					Day 2 = 0.9		
					Day 3 = 2.2		
T-2 triol	75	1.1	3.7	109.1 ± 13.9	Day 1 = 6.9	12.8	0.985
					Day 2 = 14.3		
					Day 3 = 12.5		
T-2 tetraol	75	1.2	4.0	109.0 ± 18.6	Day 1 = 19.8	17.1	0.982
					Day 2 = 9.4		
					Day 3 = 16.5		
OTA	20	0.03	0.1	113.1 ± 8.1	Day 1 = 3.1	7.2	0.987
					Day 2 = 4.8		
					Day 3 = 8.7		
OTB	20	0.6	2.0	-	-		0.988
OTα	20	0.6	2.0	-	-		0.986

## Data Availability

The original contributions presented in the study are included in the article, further inquiries can be directed to the corresponding author.

## Supplementary Materials

The following supporting information can be downloaded at: https://www.mdpi.com/article/10.3390/toxins17010034/s1, Figure S1: Distribution of zearalenone (ZEN) content (µg/kg) in kernels from sample 1, sample 2, and combined samples, with an expanded view near the legal limit for ZEN (100 µg/kg); Figure S2: Distribution of deoxynivalenol-3-glucoside (DON-3G) content (µg/kg) in kernels from sample 1, sample 2, and combined samples; Figure S3: Distribution of 15-acetyldeoxynivalenol (15-ADON) content (µg/kg) in kernels from sample 1, sample 2, and combined samples; Figure S4: Distribution of 3-acetyldeoxynivalenol (3-ADON) content (µg/kg) in kernels from sample 1, sample 2, and combined samples; Figure S5: Distribution of diacetoxyscirpenol (DAS) content (µg/kg) in kernels from sample 1, sample 2, and combined samples; Figure S6: Distribution of 15-acetoxyscirpenol (15-AS) content (µg/kg) in kernels from sample 1, sample 2, and combined samples; Figure S7: Distribution of the sum of T-2 and HT-2 toxins content (µg/kg) in kernels from sample 1, sample 2, and combined samples, with an expanded view near the legal limit for T-2+HT-2 toxins (1250 µg/kg).
