# Supplementary material for: Mapping Variability of Mycotoxins in Individual Oat Kernels from Batch Samples: Implications for Sampling and Food Safety
Source: Toxins (Basel). 2025 Jan 11;17(1):34. doi: 10.3390/toxins17010034 (PMC11768576; doi:10.3390/toxins17010034)
Supplement: Supplementary file 1 [file toxins-17-00034-s001.zip › toxins-3390293-supplementary.pdf]

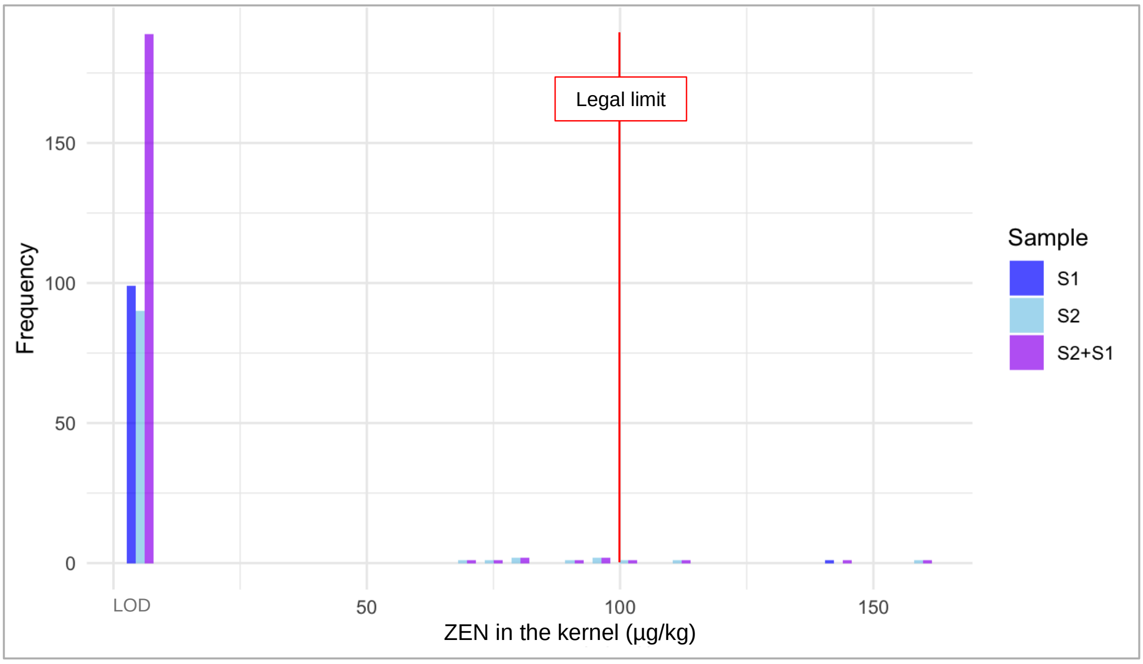

Figure S1. Distribution of zearalenone (ZEN) content (µg/kg) in kernels from sample 1, sample 2, and combined samples, with an expanded view near the legal limit for ZEN (100 µg/kg).

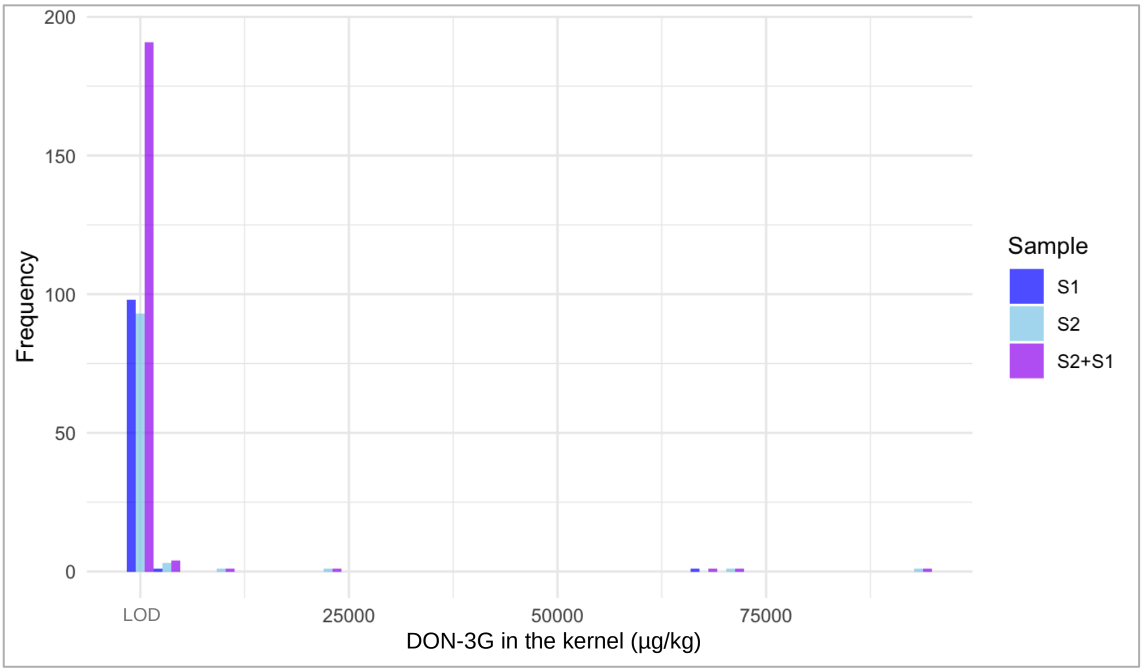

Figure S2. Distribution of deoxynivalenol-3-glucoside (DON-3G) content (µg/kg) in kernels from sample 1, sample 2, and combined samples.

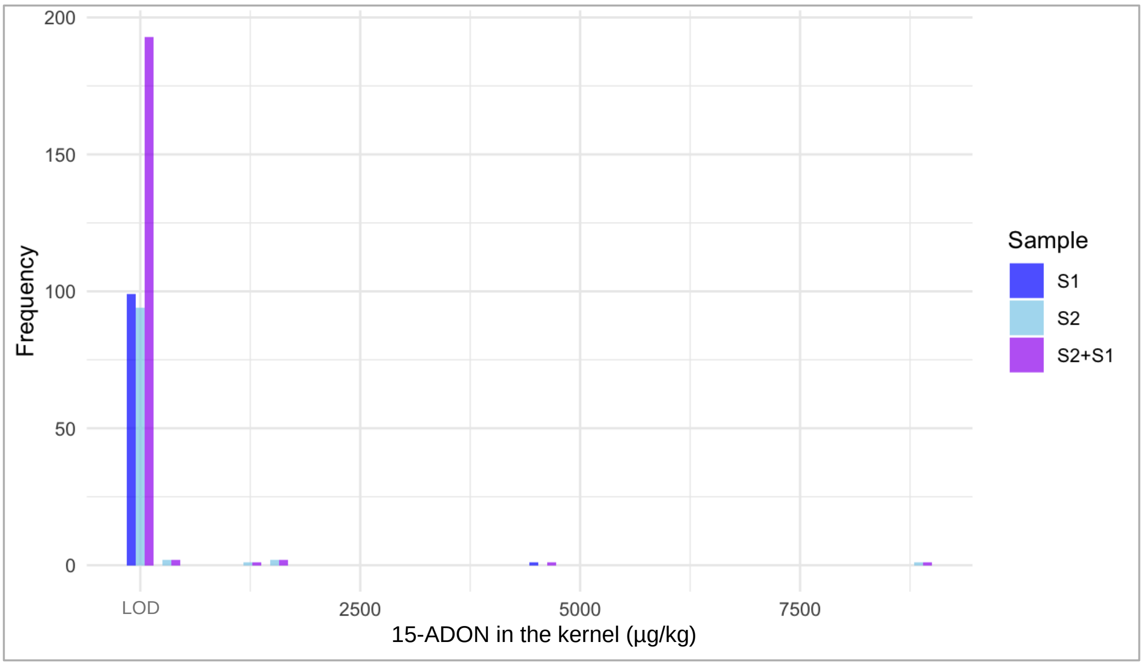

Figure S3. Distribution of 15-acetyldeoxynivalenol (15-ADON) content (µg/kg) in kernels from sample 1, sample 2, and combined samples.

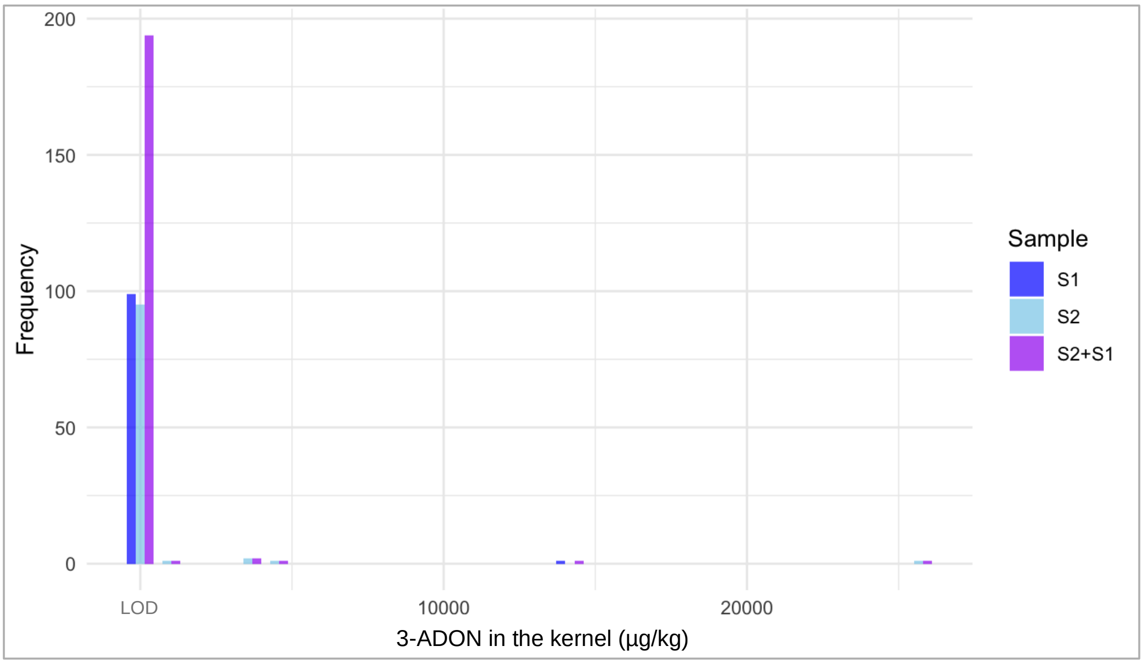

Figure S4. Distribution of 3-acetyldeoxynivalenol (3-ADON) content (µg/kg) in kernels from sample 1, sample 2, and combined samples.

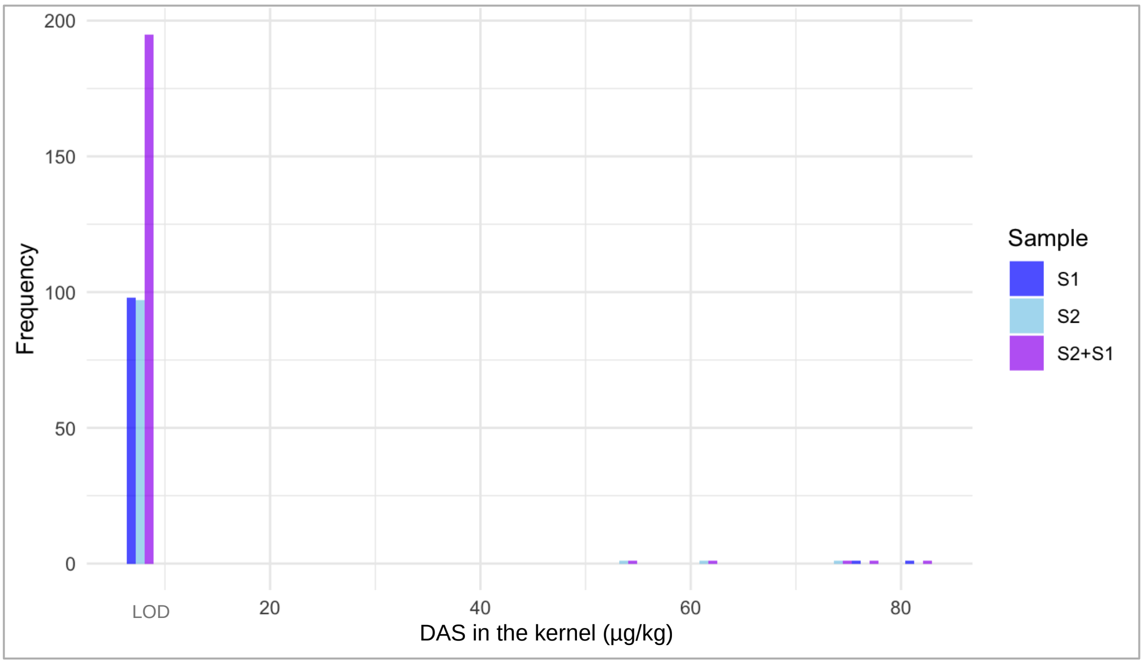

Figure S5. Distribution of diacetoxyscirpenol (DAS) content (µg/kg) in kernels from sample 1, sample 2, and combined samples.

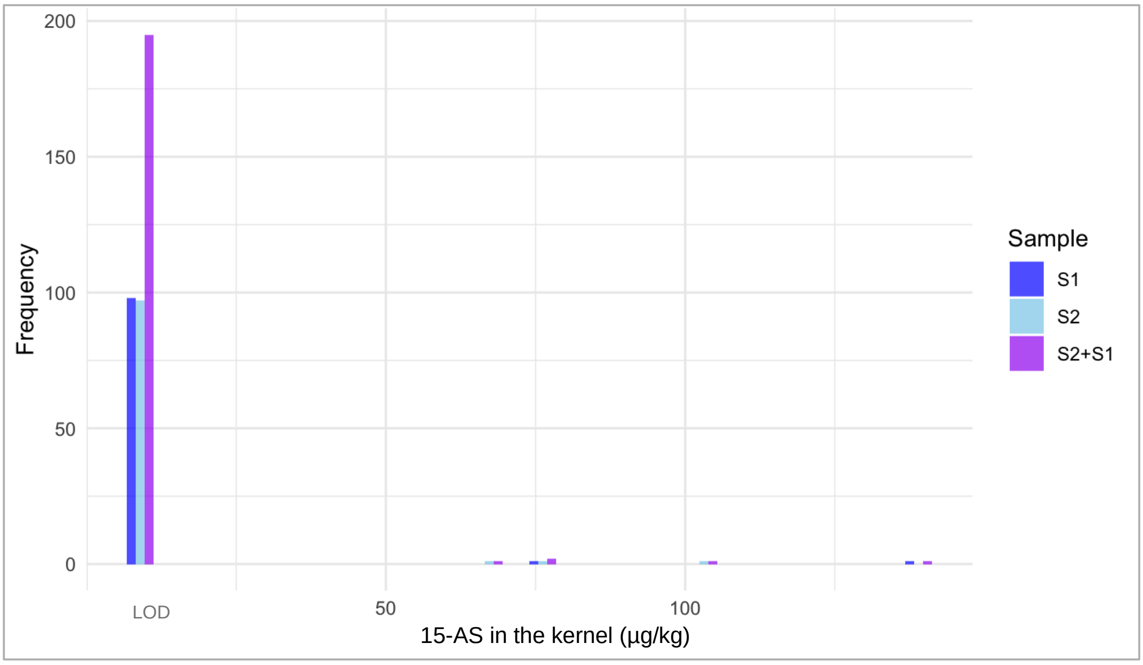

Figure S6. Distribution of 15-acetoxyscirpenol (15-AS) content (µg/kg) in kernels from sample 1, sample 2, and combined samples.

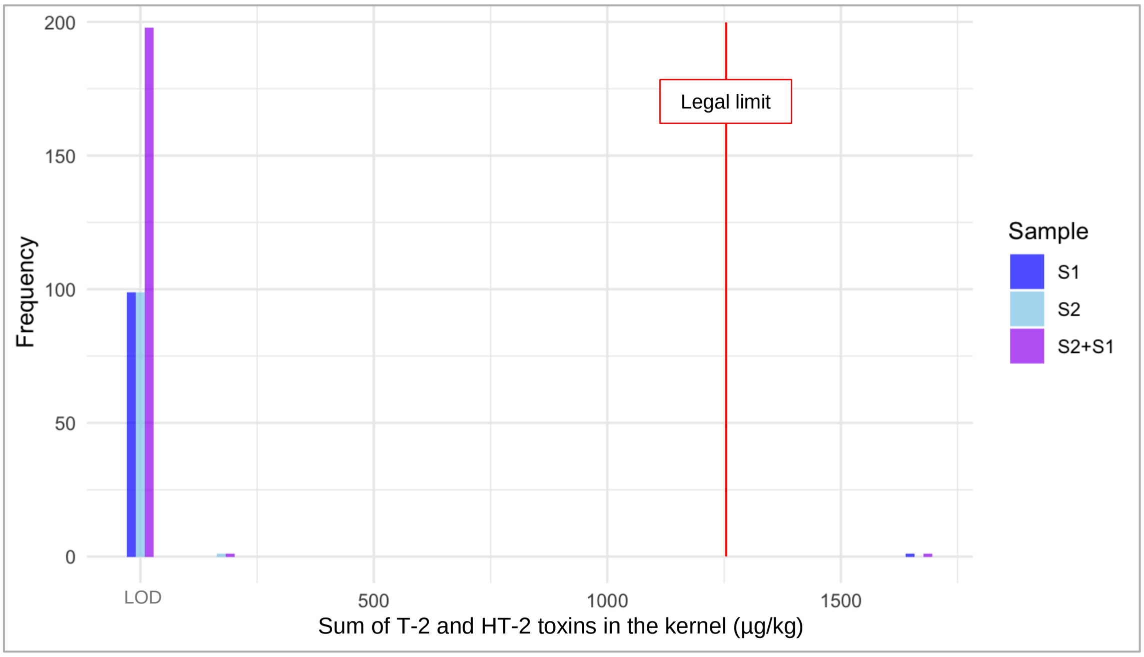

Figure S7. Distribution of the sum of T-2 and HT-2 toxins content (µg/kg) in kernels from sample 1, sample 2, and combined samples, with an expanded view near the legal limit for T-2+HT-2 toxins (1,250 µg/kg).
